# Effect of Trace Element Selenium on the Intestinal Microbial Community in Nude Mice with Colorectal Cancer

**DOI:** 10.3390/microorganisms12071336

**Published:** 2024-06-29

**Authors:** Yintong Su, Xiaohua Cai, Xingxing Fan, Jiayu Ning, Mei Shen

**Affiliations:** Department of Hygiene Inspection & Quarantine Science, Guangdong Provincial Key Laboratory of Tropical Disease Research, School of Public Health, Southern Medical University, Guangzhou 510515, China; suesuyt@163.com (Y.S.); 13421770036@163.com (X.C.); 13424081929@163.com (X.F.); ningjiayu202@163.com (J.N.)

**Keywords:** colorectal cancer, sodium selenite, selenomethionine, intestinal microbiota

## Abstract

Colorectal cancer (CRC) is the third most common cancer worldwide. The role of intestinal microbiota in carcinogenesis has also become an important research topic, and CRC is closely related to the intestinal microbiota. Selenium-containing compounds have attracted more attention as anticancer drugs as they can have minimal side effects. The purpose of this study was to determine and compare the effect of sodium selenite and selenomethionine on the microbial communities of nude mice with CRC. A CRC ectopic tumorigenesis model was established by subcutaneously injecting HCT116 cells into nude mice. The mice were then intraperitoneally injected with sodium selenite and selenomethionine for 24 days to regulate their intestinal microbiota. Compared with sodium selenite, selenomethionine resulted in a greater reduction in the richness and diversity of intestinal microbiota in nude mice with CRC, and the richness and diversity were closer to healthy levels. Selenomethionine also regulated a wider variety of flora. Additionally, sodium selenite and selenomethionine produced different microorganisms, changed function and metabolic pathways in the intestinal microbiota. Both sodium selenite and selenomethionine have certain effects on restoring the intestinal microbial diversity in nude mice with CRC, and the effect of selenomethionine is better than that of sodium selenite.

## 1. Introduction

Colorectal cancer (CRC) is the third most common cancer worldwide, and the third leading cause of cancer-related death. CRC originates in the epithelial layer of the colon or rectum, and can be caused by different factors such as heredity, diet, and the environment [1]. The relationship between intestinal microbiota and diseases is an important topic of current research. Studies examining the role of microbiota in the occurrence of cancer have suggested that microorganisms were involved in the occurrence of about 20% of cancers, especially CRC [2,3]. The level of bacteria in the colon is 1 million times higher than that in the small intestine, and the incidence of colon cancer is about 12 times higher than that in the small intestine, which suggests that the intestinal microbiota has a potential role in CRC [4]. Many studies have shown that an intestinal microbiota imbalance can adversely impact the body and result in the development of CRC [5,6]. However, the relationship between gut microbiota and CRC is not unidirectional, but rather interdependent and interactive. A study has found that after receiving treatment, the structure of CRC patients’ gut microbiota changes and gradually approaches the characteristics of normal colon microbiota [7]. Therefore, changes in the gut microbiota may be important indicators of the occurrence and development of CRC.

**Figure 1:**
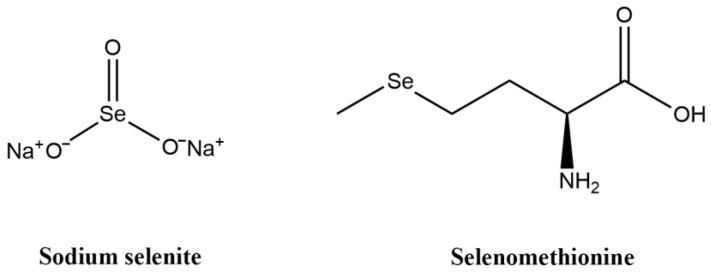
Chemical structures of sodium selenite and selenomethionine.

Selenium is an essential micronutrient for the human body. In the past 40 years, research has been mainly focused on the chemopreventative effect of selenium on cancers. Regular intake of selenium as a dietary supplement can inhibit tumor occurrence and reduce cancer risk [8,9]. High-dose selenium has attracted attention as an anticancer agent because of its high oxidation-promoting property and high selectivity for a variety of cancer cells [10]. Selenium can be divided into inorganic selenium and organic selenium, and the anticancer activity of different forms of selenium may be related to its metabolism in the body [5]. Sodium selenite (Na_2_SeO_3_), an inorganic form of selenium, has been most studied because it has been shown that it can induce cancer cell death through various mechanisms [11,12]. In food, selenium mainly exists as selenomethionine (C_5_H_11_NO_2_Se), an organic form of selenium, which is an important source of dietary selenium, a selenium supplement commonly used in clinical trials [8,9]. Clark et al. studied the effect on CRC of selenium-rich yeast in which the primary form of selenium was selenomethionine, and found that treatment with selenium-rich yeast decreased the incidence of CRC [13,14]. The anticancer effect of selenium-rich yeast may be largely attributed to selenomethionine. The chemical structures of sodium selenite and selenomethionine are displayed in Figure 1. In addition, selenium has been shown to affect the composition and colonization of intestinal microbiota, which may affect the diversity of microbiota and microbial composition [15]. Selenium can increase the relative abundance of microorganisms with potential protective effects against colitis and intestinal barrier dysfunction, such as *Turicibacter* and *Akkermansia*, to optimize the structure of the intestinal microbiota [16]. Another study has shown that selenium affects composition of the intestinal microbiota, thus affecting the selenium status of the host and the expression of selenoproteome [17].

Extensive in vitro and in vivo experimental studies have been conducted to delve into the mechanisms underlying selenium’s impact on CRC, revealing its inhibitory effects on CRC cells and suppression of CRC tumor development [18,19,20]. However, no studies have examined how the intestinal microbiota is affected by inorganic selenium and organic selenium during the treatment of CRC. Studying the changes in intestinal microbiota can help to gain a deeper understanding of the mechanism of selenium’s effect on CRC. Therefore, the purpose of this study was to examine the effects of inorganic selenium and organic selenium (sodium selenite and selenomethionine, respectively) on the intestinal microbial community using a nude mice CRC model. We expect the results to help in understanding the effect of selenium on the intestinal microbiota in the treatment of CRC.

## 2. Materials and Methods

### 2.1. Reagents

The L-selenomethionine was purchased from CSNpharm (Shanghai, China). The sodium selenite was purchased from Tianjin Beilian Fine Chemicals Development (Tianjin, China). The phosphate-buffered saline (PBS) was purchased from Dewei Biological Technology (Guangzhou, China). The DNA extraction kit was purchased from Omega Bio-Tek (Norcross, GA, USA). The FastPfu Polymerase was purchased from TransGen Biotech (Beijing, China). The AxyPrep DNA Gel Extraction Kit was purchased from Axygen Scientific (Union City, CA, USA). The rapid DNA library prep kit was purchased from Bioo Scientific (Austin, TA, USA). The MiSeq Reagent Kit v3 and NovaSeq Reagent Kits were purchased from Illumina (San Diego, CA, USA).

### 2.2. Cells and Animals

The HCT116 cell lines were obtained from the Cancer Research Institute, School of Basic Medical Sciences, Southern Medical University. The twenty-four 4-week-old SPF BALB/c male nude mice, weighing 10 to 15 g, were purchased from Guangdong Medical Laboratory Animal Center. The temperature of the feeding environment was kept at 23 ± 3 °C, the humidity was kept at 50 ± 5%, one-way flow ventilation was used in the room, the illumination period was 12 h light/12 h dark, and the mice could drink water and eat freely. The experiment complied with the ARRIVE guidelines and all animal care and husbandry procedures were performed under the National Research Council’s Guide for the Care and Use of Laboratory Animals. The study was approved by the Experimental Animal Ethics Committee of the Southern Medical University.

### 2.3. Experimental Design

**Figure 2:**
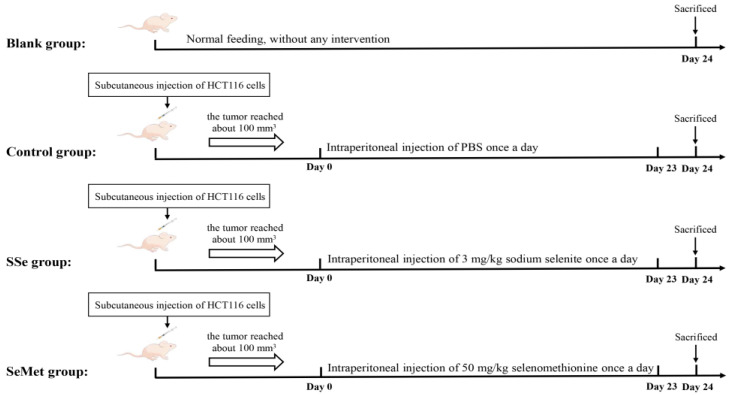
Schematic diagram of the experimental design.

The BALB/c nude mice were adaptively fed for 3 days, and then they were randomly divided into 4 groups (*n* = 6 per group): the Blank group, Control group, SSe group and SeMet group. The Blank group were given a normal diet and drank water freely, without any intervention. For the subcutaneous xenograft model, HCT116 cells in the logarithmic growth phase were collected and 0.1 mL of HCT116 cells (adjusted to a cell density of 1 × 10^7^ cells/mL) were subcutaneously inoculated in the right lower groin of the mice. Tumor growth was observed every day, the long diameter (L) and short diameter (W) of the tumor was measured and the tumor volume was calculated (tumor volume = 1/2 L × W^2^). When the tumor reached about 100 mm^3^, the interventions were as follows: (1) mice in the Control group were injected intraperitoneally with PBS (0.01 mL/kg body weight) once a day; (2) mice in the SSe group were injected intraperitoneally with sodium selenite (3 mg/kg body weight) once a day; (3) mice in the SeMet group were injected intraperitoneally selenomethionine (50 mg/kg body weight) once a day. The interventions were performed for 24 days. The timeline of the experimental design is shown in Figure 2.

### 2.4. H&E Staining and Immunohistochemical Staining

Tumor tissues were dissected from the mice and fixed in a fixative solution. After 24 h, the tissues were taken out of the fixative solution, finely trimmed, and placed in an embedding frame, dehydrated, dipped in wax, and finally embedded in paraffin for sectioning. After that, the tissues were dewaxed and hydrated, stained with hematoxylin and eosin, dehydrated, and sealed. Image acquisition and analysis were performed using a microscope.

For immunohistochemical (IHC) staining, tumor tissues were sliced, dewaxed, and dehydrated, and then antigen repair was performed for 15 min. The slices were placed in 3% hydrogen peroxide solution at room temperature, away from light, for 25 min, and then were sealed with 3% BSA at room temperature for 30 min. After sealing, the slices were incubated with anti-Ki67 primary antibody at 4 °C overnight. After washing three times in PBS, the slices were incubated with the secondary antibody bound to HRP at room temperature for 50 min. The slides were treated with diaminobenzidine (DAB) to visualize the color, followed by staining with hematoxylin for 3 min, dehydration, and sealing. Image acquisition and analysis were performed using a microscope.

### 2.5. Collection of Fecal Samples, DNA Extraction, and 16S rRNA Sequencing

The mice to be sampled were put into a clean cage with the floor covered with sterile filter paper, and fecal samples were collected immediately after defecation. New filter paper was used for the sampling of each mouse. Three to five fecal samples were collected from each nude mouse, put into a sterile centrifuge tube, frozen in liquid nitrogen, and stored at −80 °C, and sent for analysis packed with dry ice. The total DNA of the microbial community was extracted according to the instructions of the E.Z.N.A.^®^ soil DNA kit, the quality of the extracted DNA was tested by 1% agarose gel electrophoresis, and the concentration and purity of the DNA were determined by NanoDrop2000 (Waltham, MA, USA). High-throughput sequencing on a Meggie platform was performed by Shanghai Meggie Biomedical Technology Co., Ltd. (Shanghai, China).

In order to analyze the composition of the microbial community, the V3-V4 region of the 16S rRNA gene was selected for amplification and sequencing. Universal primers were upstream primer 338F and downstream primer 806R. The PCR reaction system was as follows: 4 μL of 5×*TransStart* FastPfu buffer, 2.5 mM dNTPs 2 μL, 0.8 μL of upstream primer (5 uM), 0.8 μL of downstream primer (5 uM), 0.4 μL of *TransStart* FastPfu DNA polymerase, 0.2 μL of BSA, 10 ng of template DNA and 20 μL of ddH_2_O. The amplification procedure was as follows: pre-denaturation at 95 °C for 3 min, 27 cycles (denatured at 95 °C for 30 s, annealed at 55 °C for 30 s, and extended at 72 °C for 45 s), stably extended at 72 °C for 10 min, and stored at 10 °C. The PCR instrument was ABI Gene Amp^®^9700 PCR themocycler (ABI, Oakland, CA, USA).

All sequencing reads were screened, and the original sequencing sequence was controlled by fastp software (version 0.20.0), and spliced by FLASH software (version 1.2.7) to remove low-quality bases. The UPARSE software (version 7.1) was used for OTU cluster and chimera removal, according to the similarity of 97%. Each sequence was annotated by RDP classifier and was compared with the database of Silva 16S rRNA. The comparison threshold was set at 70%. The 16S rRNA sequencing data of the mice fecal microbiota were deposited in the Sequence Read Archive (SRA) database and can be accessed by the accession number PRJNA1056856.

### 2.6. Microbiome Data Analysis

Alpha diversity among the groups was compared using Sobs index and Shannon index. Beta diversity comparison among the groups was carried out by PLS-DA analysis (partial least squares discriminant analysis). Based on the obtained community abundance data. LEfSe (linear discriminant analysis coupled with effect size analysis) performed linear discriminant analysis (LDA) on samples according to different grouping conditions based on taxonomic composition to find out the biomarkers. PICRUSt2 (version 2.2.0) with the Clusters of Orthologous Groups of proteins (COG) database and the Kyoto Encyclopedia of Genes and Genomes (KEGG) database was used as a reference to infer the metabolism functions of intestinal microbiota.

### 2.7. Statistical Analyses

Data were expressed as mean deviation. The Kruskal–Wallis H test and Wilcoxon rank sum test were used to analyze the differences between groups. The test level was set as α = 0.05, and a value of *p* < 0.05 was considered to indicate a statistically significant difference.

## 3. Results

### 3.1. Selenium Inhibited the Development of CRC Tumors

**Figure 3:**
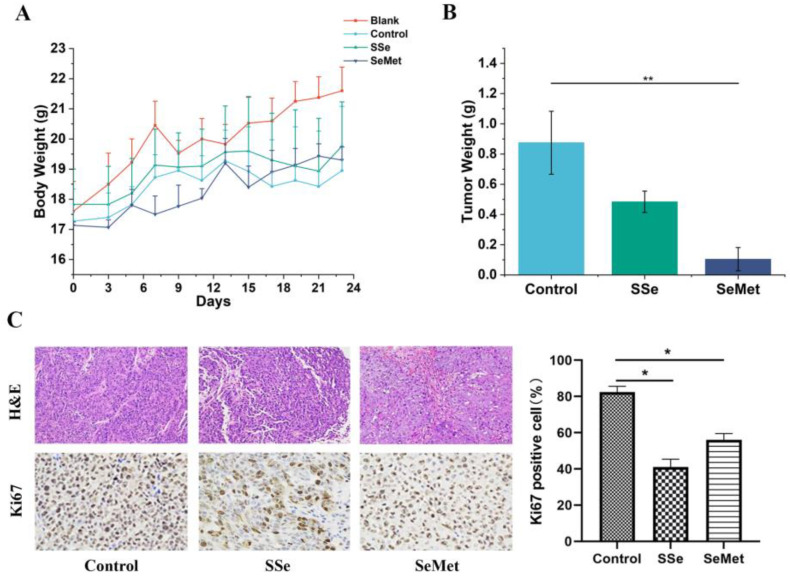
Inhibitory effects of sodium selenite and selenomethionine on nude mice with CRC. (**A**) Trend chart of body weight in each group of mice. (**B**) Tumor weight of mice in Control group, SSe group and SeMet group. (**C**) H&E staining results of tumor tissues (×200) and the expression of Ki67 in tumor tissues analyzed by IHC staining (×400) of mice in Control group, SSe group and SeMet group. (* indicating *p* < 0.05, ** indicating *p* < 0.01).

Compared with the Blank group, the mice in the Control group, SSe group and SeMet group had decreased weight (Figure 3A). The body weight of CRC nude mice treated with sodium selenite and selenomethionine showed no difference compared to the Control group. Our results indicated that the tumor weight was greater in the Control group than in the SSe group and SeMet group (Figure 3B). Moreover, selenomethionine treatment significantly reduced the tumor weight (*p* < 0.01). Compared with the Control group, sodium selenite and selenomethionine significantly inhibited the proliferation of tumor cells (Figure 3C).

### 3.2. Sequencing Data

A total of 1,359,987 trimmed sequences were obtained from four groups. The average sequence length was 422 bp. The sequencing results of each group were shown in Table 1.

### 3.3. The Effect of Selenium on Intestinal Microbial Richness and Diversity of CRC Nude Mice

**Figure 4:**
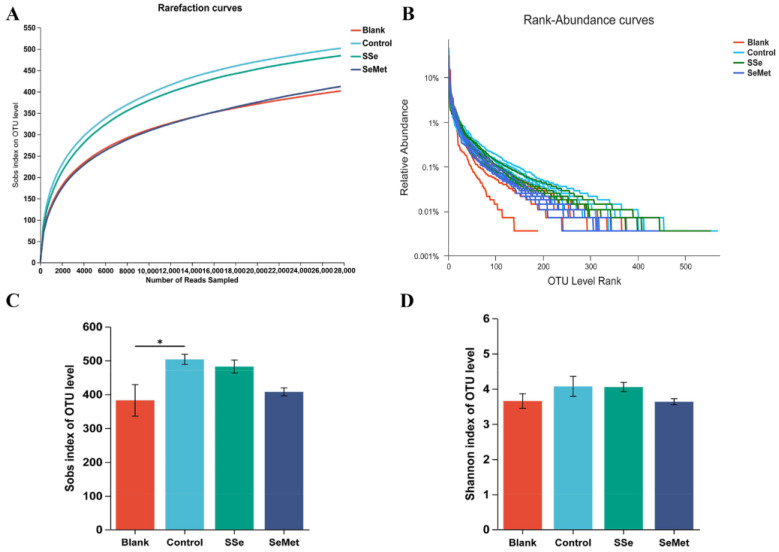
Alpha diversity analysis. (**A**) Rarefaction curves were constructed using Sobs indices. (**B**) Rank–abundance curves on OTU level. (**C**) Measures of richness using Sobs index. (**D**) Measures of diversity using Shannon index. (* indicating *p* < 0.05).

The dilution curve of samples in each group reached a stable state after 28,000 readings, which indicated that the curves had reached the plateau phase (Figure 4A). The rank abundance curves also became stable, indicating that the species distribution was uniform (Figure 4B). This result showed that the sequencing data were reasonable.

The Sobs index (Figure 4C) and Shannon index (Figure 4D) were used to evaluate the richness and diversity of the intestinal microbiota. Sobs index and Shannon index in the Control group were higher than those in Blank group, and the Sobs index in the Blank group and Control group was statistically significant (*p* < 0.05). This result indicated a significant increase in the richness and diversity of intestinal microbiota in the CRC nude mice. The Sobs index and Shannon index in the SSe group and the SeMet group were lower than those in the Control group, but there were no significant differences. The Sobs index and Shannon index in the Control group, SSe group, and SeMet group were higher than those in the Blank group, but the values in the SeMet group were close to those in the Blank group, indicating that the richness and diversity of intestinal microbiota in the CRC nude mice treated with SeMet were closer to healthy levels.

### 3.4. The Effect of Selenium on Intestinal Microbial Structure of CRC Nude Mice

**Figure 5:**
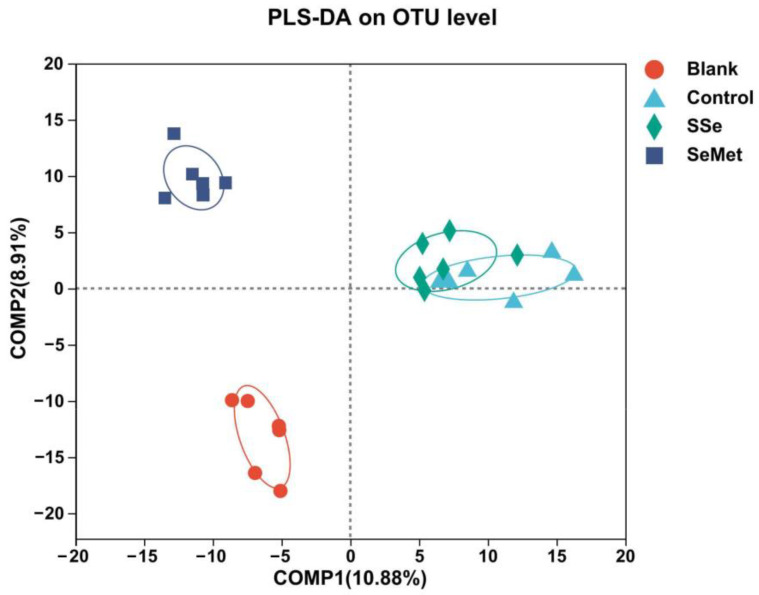
Partial least squares discriminant analysis (PLS-DA) analysis on the OTU level.

The partial least squares discriminant analysis diagram (Figure 5) showed that the Blank group, Control group, SSe group, and SeMet group formed four obviously separated areas, indicating that there were significant differences in the composition of intestinal microbiota among the groups. In addition, the discrete state of sample points distribution showed that the Blank group and SeMet group were distributed in the left quadrant, and the Control group and SSe group were distributed in the right quadrant, with obvious overlap.

### 3.5. The Effect of Selenium on Intestinal Microbial Composition of CRC Nude Mice

**Figure 6:**
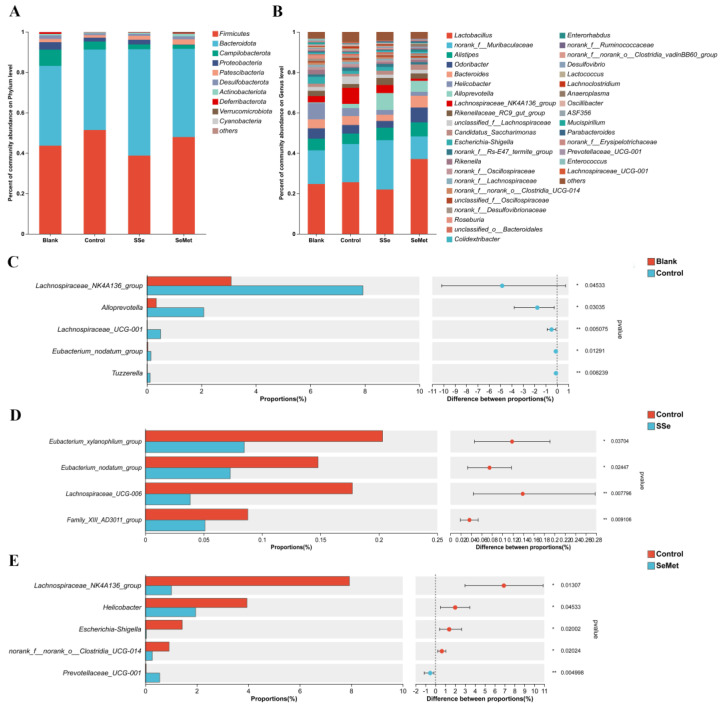
The effect of sodium selenite and selenomethionine on intestinal microbiota composition. (**A**) Relative abundance at the phylum level in fecal microbiota of each group. (**B**) Relative abundance at the genus level in fecal microbiota of each group. (**C**) Difference analysis between Blank group and Control group. (**D**) Difference analysis between Control group and SSe group. (**E**) Difference analysis between Control group and SeMet group. (* indicating *p* < 0.05, ** indicating *p* < 0.01).

The composition of intestinal microbiota of the nude mice in each group was analyzed at the phylum (Figure 6A) and genus levels (Figure 6B). At the phylum level, the dominant phyla of the intestinal microbiota in the four groups were *Firmicutes* and *Bacteroidota*. At the genus level, the dominant genera of the intestinal microbiota in the four groups were *Lactobacillus* and *norank_f__Muribaculaceae*. A difference analysis on the abundances of intestinal microbiota was performed (Figure 6C–E). Compared with the healthy nude mice, the abundance of some intestinal microbiota changed after tumorigenesis. Compared with the Blank group, the abundances of *Lachnospiraceae_NK4A136_group*, *Alloprevotella*, *Lachnospiraceae_UCG-001*, *Eubacterium_nodatum_group*, and *Tuzzerella* in the Control group increased (*p* < 0.05). After the nude mice with CRC were treated with sodium selenite or selenomethionine, the abundances of some intestinal microbiota in the SSe group and the SeMet group changed. Compared with the Control group, the SSe group decreased the abundances of *Eubacterium_xylanophilum_group*, *Eubacterium_nodatum*_*group*, *Lachnospiraceae_UCG-006*, and *Family_XIII_AD3011_group* (*p* < 0.05); the SeMet group saw a decrease in the abundances of *Lachnospiraceae_NK4A136_group*, *Helicobacter*, *Escherichia-Shigella* and *norank_f__norank_o__Clostridia_UCG-014*, and an increase in the abundance of *Prevotellaceae_UCG-001* (*p* < 0.05).

### 3.6. Linear Discriminant Analysis (LDA) Effect Size (LEfSe)

**Figure 7:**
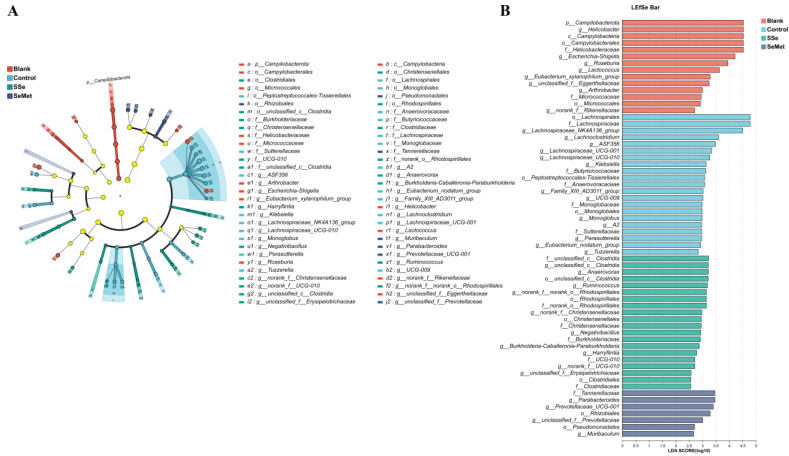
Cladogram of significant difference between groups. (**A**) Cladogram constructed using the linear discriminant analysis effect size (LefSe) method to indicate the phylogenetic distribution of bacteria that were remarkably enriched between each group. Yellow circles indicate species with no significant differences. (**B**) Linear discriminant analysis (LDA) scores represent the gut bacteria which were of important biological significance in each group.

In order to identify the different intestinal microbiota in the groups, the samples were analyzed by LDA (Figure 7). According to the taxonomic analysis, the LDA score of the intestinal microbiota of 1 phylum, 1 class, 11 orders, 14 families, and 35 genera was 2 (*p* < 0.05, Table S1). At the genus level, *Helicobacter*, *Escherichia-Shigella*, *Roseburia*, *Lactococcus*, *Eubacterium_xylanophilum_group*, *unclassified_f__Eggerthellaceae*, *Arthrobacter* and *norank_f__Rikenellaceae* were significantly enriched in the Blank group. *Lachnospiraceae_NK4A136_group*, *Lachnoclostridium*, *ASF356*, *Lachnospiraceae_UCG-001*, *Lachnospiraceae_UCG-010*, *Klebsiella*, *Family_XIII_AD3011_group*, *UCG-009*, *Monoglobus*, *A2*, *Parasutterella*, *Eubacterium_nodatum_group* and *Tuzzerella* were significantly enriched in the Control group. *Unclassified_c__Clostridia*, *Anaerovorax*, *Ruminococcus*, *norank_f__norank_o__Rhodospirillales*, *norank_f__Christensenellaceae*, *Negativibacillus*, *Burkholderia-Caballeronia-Paraburkholderia*, *Harryflintia*, *norank_f__UCG-010* and *unclassified_f__Erysipelotrichaceae* were significantly enriched in the SSe group. *Parabacteroides*, *Prevotellaceae_UCG-001*, *unclassified_f__Prevotellaceae* and *Muribaculum* were significantly enriched in the SeMet group.

### 3.7. Microbiota Function Prediction

**Figure 8:**
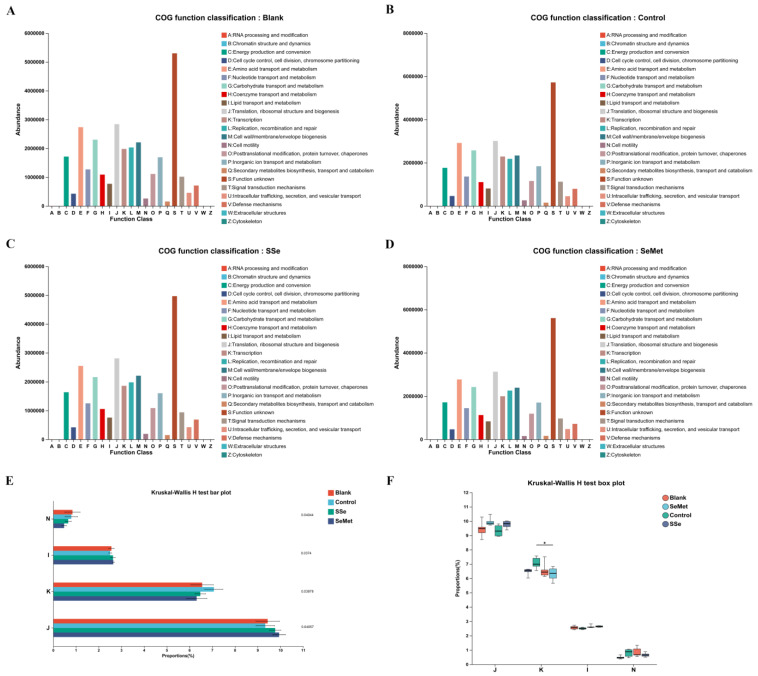
Bar chart of the Clusters of Orthologous Genes (COG) function classification in (**A**) Blank group, (**B**) Control group, (**C**) SSe group and (**D**) SeMet group. (**E**) Bar chart and (**F**) box plot of COG functions with differences in each group. N: cell motility; I: lipid transport and metabolism; K: transcription; J: translation, ribosomal structure and biogenesis. (* indicating *p* < 0.05).

**Figure 9:**
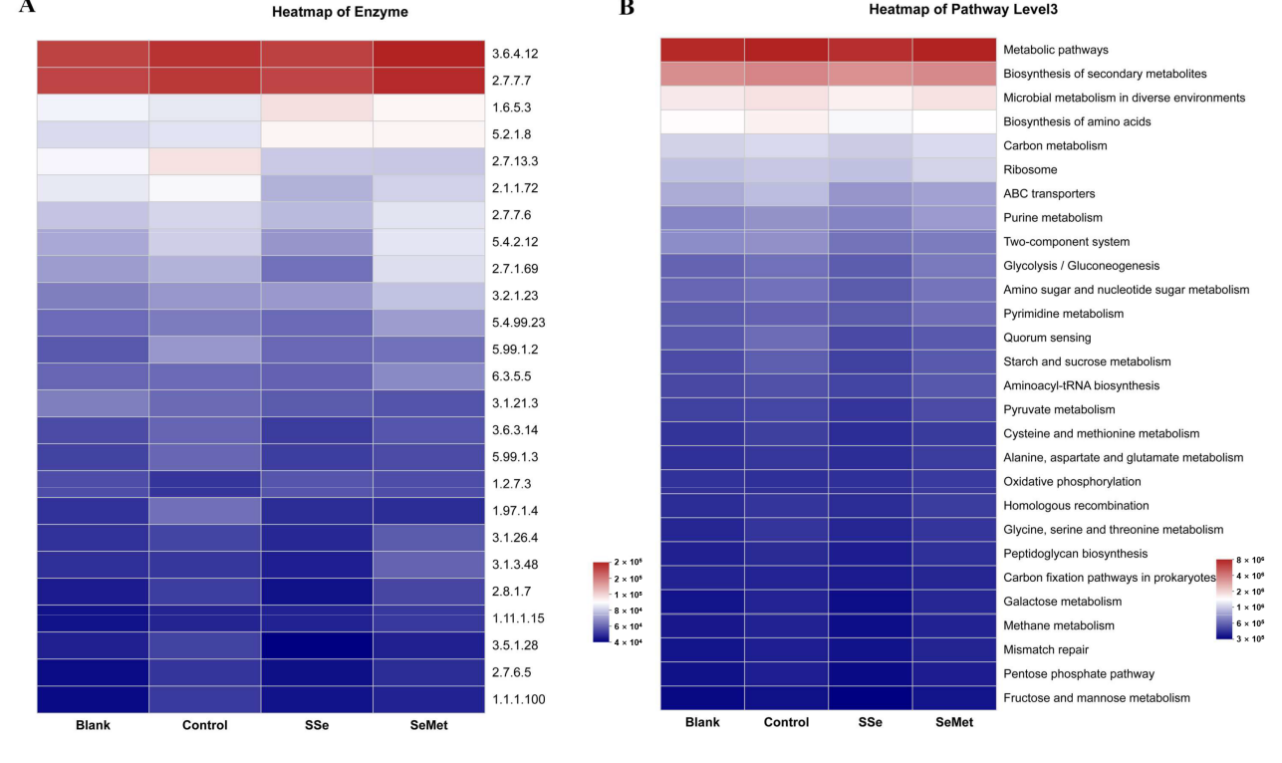
Functional analysis of (**A**) enzyme and (**B**) Kyoto Encyclopedia of Genes and Genomes (KEGG) pathway level3 based on KEGG database.

To study the functional and metabolic changes of the intestinal microbial communities, we compared the measured sequences with the GOG database and KEGG database. Through referring to COG database (Figure 8), 23 pathways involved in the metabolism of intestinal microbiota were predicted, including RNA processing and modification, chromatin structure and dynamics, energy production and conversion, etc. The COG potential functional annotation results also showed that there were significant differences in the functions such as cell motility (*p* = 0.0404), lipid transport and metabolism (*p* = 0.0374), transcription (*p* = 0.0388) and translation, ribosomal structure and biogenesis (*p* = 0.0406) among the groups. Meanwhile, there was a significant difference in transcription between the Control group and SeMet group (*p* < 0.05). Moreover, the KEGG potential functional annotation results (Figure 9) showed that the histidine kinase (EC 2.7.13.3) and biosynthesis of amino acids were significantly enriched in the Control group. Ubiquinone reductase (EC 1.6.5.3) and peptidylprolyl isomerase (EC 5.2.1.8) were significantly enriched in the SSe group and SeMet group.

## 4. Discussion

In this study, the effects of sodium selenite and selenomethionine in CRC nude mice were analyzed, and their mechanisms of action were examined based on intestinal microbiota. Our results showed that sodium selenite and selenomethionine could reduce the weight of CRC tumors and inhibit the proliferation of CRC tumor cells. It has been reported that the overexpression of selenium-binding protein 1 (SELENBP1) in HCT116 cells not only suppressed cell proliferation, increased apoptotic cell death, and decreased cell migration, but also inhibited tumor growth and angiogenesis [21,22]. These results indicate that selenium can inhibit the development of CRC tumors.

The imbalance of intestinal microbiota in nude mice with CRC is related to an increase in many potential pathogens. The differences in the Sobs index and Shannon index between healthy mice and CRC nude mice indicated a high richness and diversity in CRC nude mice. The findings suggest that the occurrence of CRC has a certain impact on the species and quantity of intestinal microbiota in nude mice, indicating that CRC leads to the overgrowth of some harmful intestinal microbiota, which increases the richness and variety of intestinal microbiota. The abundances of intestinal microbiota between healthy nude mice and mice in the Control group were compared, and the abundances of *Lachnospiraceae_NK4A136_group* and *Alloprevotella* were higher in the Control group. *Lachnospiraceae_NK4A136_group* has been reported to be positively associated with pathological features of colitis [23]. *Anaeroplasma* is an opportunistic pathogen in these genera, and has been reported to be associated with intestinal inflammation [24]. A recent cohort study of CRC patients in Hainan Province, China, showed that some species of *Alloprevotella* were significantly enriched in CRC patients [25]. We also found that the abundance of *Alloprevotella* was increased in nude mice with CRC. These findings indicate that CRC promotes the growth of harmful microorganisms in the intestine of nude mice.

After treatment with sodium selenite or selenomethionine, the structure and composition of intestinal microbiota in the nude mice with CRC changed. Sodium selenite and selenomethionine reduced the richness and diversity of intestinal microbiota in CRC nude mice. Compared with the CRC nude mice treated with sodium selenite, the richness and diversity of intestinal microbiota in mice treated with selenomethionine were lower, and were closer to those in healthy nude mice. In addition, PLS-DA analysis also confirmed that the intestinal microbiota of healthy nude mice was similar to that of CRC nude mice treated with selenomethionine. Therefore, compared with CRC nude mice treated with sodium selenite, the intestinal microbiota of the mice treated with selenomethionine was closer to that of healthy nude mice. Yang et al. inoculated nude mice with the human CRC SW480 cell line and found that a high concentration of sodium selenite and selenomethionine had similar anticancer effects [19]. In this study, it was found that sodium selenite and selenomethionine had a certain therapeutic effect on CRC from the perspective of intestinal microbiota, and the effect of selenomethionine on CRC was better than that of sodium selenite. Selenomethionine is higher in bioavailability and less toxic than sodium selenite, presumably because its incorporation into tissue proteins removes a portion from the circulatory system [26]. One study has demonstrated that several inorganic and organic selenocompounds were metabolized to selenomethionine by the intestinal microbiota and that selenomethionine was incorporated into bacterial proteins, leading to the accumulation of selenomethionine-containing proteins that serve as a selenium reservoir for the host animal [27]. Therefore, in comparison to sodium selenite, selenomethionine exhibits a more moderate biological activity, and its ability to be absorbed by bacteria and integrated into their proteins indicates that selenomethionine might exert a more direct influence on the composition and functionality of the intestinal microbiota, ultimately leading to a more effective modulation of the intestinal microbiota.

We found that treatment with sodium selenite and selenomethionine resulted in a decrease in the abundances of harmful bacteria and an increase in the abundances of beneficial bacteria. Sodium selenite could reduce the abundance of *Eubacterium_xylanophilum_group*. A higher relative abundance *Eubacterium_xylanophilum_group* was found in DSS-induced colitis mice [28]. Selenomethionine could reduce the abundances of the harmful bacteria *Helicobacter, Lachnospiraceae_NK4A136_group* and *Escherichia-Shigella*. It has been reported that *Helicobacter* were increased in CRC tissue [29]. Shao et al. reported that *Lachnospiraceae_NK4A136_group* is related to inflammatory bowel disease [30]. *Escherichia-Shigella* is a Gram-negative bacterium that can produce bacterial toxins such as lipopolysaccharides [31]. It has been reported that *Escherichia-Shigella* was closely related to pro-inflammatory responses, and its abundance was positively correlated with pro-inflammatory factors including IL-6 and NLRP3 [32,33]. In addition, obese CRC patients exhibit a significantly higher abundance of *Escherichia-Shigella* compared to healthy individuals and non-obese CRC patients, suggesting a potential link between this bacterium and the development of CRC [34]. We also found that selenomethionine could increase the abundance of *Prevotellaceae_UCG-001*. *Prevotellaceae_UCG-001*, a bacterium that produces SCFA, was found to have a lower relative abundance in DSS-induced colitis mice [35,36]. Therefore, we speculate that selenomethionine may regulate the intestinal microbiota of CRC nude mice by regulating inflammation-related bacteria.

The LEfSe method was used to identify biomarkers in each group at each level. At the genus level, *Roseburia* and *Eubacterium_xylanophilum_group* were characteristic intestinal microorganisms of healthy nude mice. *Roseburia* and *Eubacterium_xylanophilum_group* are intestinal microorganisms that produce butyrate [37]. Sufficient butyrate can prevent CRC by reducing oxidative damage to DNA, inducing apoptosis of DNA-damaged cells, inhibiting the growth of tumor cells, and reducing the activity of co-oncogenes [38,39]. Liang et al. reported that *Lachnoclostridium* is a fecal bacterial marker of CRC [40]. Our results also showed that *Lachnoclostridium* was significantly enriched in the Control group. Moreover, we found that *Lachnospiraceae_NK4A136_group*, *ASF356*, *Lachnospiraceae_UCG-001*, *Lachnospiraceae_UCG-010*, *Klebsiella, Family_XIII_AD3011_group*, *UCG-009*, *Monoglobus*, *A2*, *Parasutterella*, *Eubacterium_nodatum_group* and *Tuzzerella* were characteristic the intestinal microorganisms of nude mice with CRC. After treatment with sodium selenite and selenomethionine, the characteristic intestinal microorganisms of nude mice with CRC were slightly changed. We found nine characteristic intestinal microorganisms including *Unclassified_c__Clostridia*, *Anaerovorax*, *Ruminococcus*, *norank_f__norank_o__Rhodospirillales*, *norank_f__Christensenellaceae*, *Burkholderia-Caballeronia-Paraburkholderia*, *Harryflintia*, *norank_f__UCG-010* and *unclassified_f__Erysipelotrichaceae* in nude mice with CRC treated with sodium selenite, and four characteristic intestinal microorganisms including *Parabacteroides*, *Prevotellaceae_UCG-001*, *unclassified_f__Prevotellaceae* and *Muribaculum* in nude mice with CRC treated with selenomethionine.

Through functional prediction, we found some changes in certain COG functions and KEGG pathways in each group. The COG annotation analysis and KEGG pathways showed that the dysbiosis of intestinal microbiota in nude mice with CRC is associated with several pathways, including histidine kinase and biosynthesis of amino acids. Histidine kinase plays an important role in tumor proliferation, invasion, metastasis, and poor prognosis by regulating intracellular histidine phosphorylation levels [41]. After treatment with sodium selenite and selenomethionine, changes in the intestinal microbiota in nude mice with CRC are associated with ubiquinone reductase and peptidylprolyl isomerase, which provided direction for discovering the potential role and mechanism of intestinal microbiota in the action of sodium selenite and selenomethionine on nude mice with CRC. The functional changes in different groups produce tumorigenic or protective effects and may serve as targets for the next treatment of CRC.

## 5. Conclusions

Our study showed that selenium can inhibit the development of CRC tumors and regulate the species and quantity of intestinal microbiota. Both sodium selenite and selenomethionine reduced the richness and diversity of intestinal microbiota, increased beneficial bacteria and reduced harmful bacteria in the intestine of nude mice with CRC. Compared with sodium selenite, selenomethionine resulted in a greater reduction in the richness and diversity of intestinal microorganisms in nude mice with CRC, and the richness and diversity were closer to that of healthy nude mice. Selenomethionine also regulated a wider variety of flora with respect to promoting beneficial bacteria and reducing the number of harmful bacteria. In conclusion, both sodium selenite and selenomethionine have certain effects on restoring the intestinal microbial diversity in nude mice with CRC, and the effect of selenomethionine is better than that of sodium selenite. This study will aid the understanding of the changes and effects of intestinal microbiota in the use of selenium in the treatment of CRC.

## Figures and Tables

**Table 1:** Statistics of sample sequencing results.

Group	Seq_Num	Base_Num	Mean_Length	OTU_Num
Blank	389,981	164,773,284	422	772
Control	286,454	120,141,670	420	754
SSe	293,661	123,729,704	421	768
SeMet	389,891	164,773,284	423	700

## Data Availability

All other data are available from the authors upon reasonable request.

## Supplementary Materials

The following supporting information can be downloaded at: https://www.mdpi.com/article/10.3390/microorganisms12071336/s1, Table S1: The result of linear discriminant analysis (LDA). The nonparametric factorial Kruskal–Wallis H test was used to find species with significant differences from the phylum to genus level (*p* < 0.05), in which the value of LDA was greater than 2. p, c, o, f and g represent the taxonomic level, which were phylum, class, order, family and genus, respectively.
