# Supplementary material for: Effect of Trace Element Selenium on the Intestinal Microbial Community in Nude Mice with Colorectal Cancer
Source: Microorganisms. 2024 Jun 29;12(7):1336. doi: 10.3390/microorganisms12071336 (PMC11279152; doi:10.3390/microorganisms12071336)
Supplement: Supplementary file 1 [file microorganisms-12-01336-s001.zip › Table S1.pdf]

Table S1

The result of linear discriminant analysis (LDA). The nonparametric factorial Kruskal-Wallis (KW) sum rank test was used to find species with significant differences from the phylum to genus level ($p < 0.05$), in which the value of LDA was greater than 2. p, c, o, f and g represent the taxonomic level, which were phylum, class, order, family and genus, respectively.

**Species name**	**Group**	**Mean**	**LDA-value**	***p*-value**
p__Firmicutes.c__Clostridia.o__unclassified_c__Clostridia.f__unclassified_c__Clostridia	SSe	1.82	3.22	0.015
p__Bacteroidota.c__Bacteroidia.o__Bacteroidales.f__Prevotellaceae.g__Prevotellaceae_UCG-001	SeMet	3.74	3.40	0.019
p__Firmicutes.c__Clostridia.o__Lachnospirales.f__Lachnospiraceae.g__Roseburia	Blank	4.23	3.95	0.011
p__Firmicutes.c__Clostridia.o__Peptostreptococcales-Tissierellales	Control	3.44	3.08	0.001
p__Firmicutes.c__Clostridia.o__Lachnospirales.f__Lachnospiraceae.g__Lachnospiraceae_UCG-001	Control	3.70	3.35	0.002
p__Firmicutes.c__Clostridia.o__Christensenellales.f__Christensenellaceae.g__norank_f__Christensenellaceae	SSe	1.89	2.97	0.016
p__Firmicutes.c__Clostridia.o__Oscillospirales.f__Ruminococcaceae.g__Negativibacillus	SSe	3.22	2.93	0.000
p__Firmicutes.c__Clostridia.o__Oscillospirales.f__Butyricicoccaceae.g__UCG-009	Control	3.25	3.01	0.000
p__Bacteroidota.c__Bacteroidia.o__Bacteroidales.f__Prevotellaceae.g__unclassified_f__Prevotellaceae	SeMet	3.00	3.01	0.003
p__Firmicutes.c__Clostridia.o__Lachnospirales.f__Lachnospiraceae.g__Tuzzerella	Control	3.08	2.84	0.018
p__Proteobacteria.c__Alphaproteobacteria.o__Rhizobiales	SeMet	2.32	3.29	0.006
p__Firmicutes.c__Clostridia.o__Lachnospirales.f__Lachnospiraceae.g__Lachnospiraceae_NK4A136_group	Control	4.90	4.50	0.007
p__Campilobacterota.c__Campylobacteria.o__Campylobacterales.f__Helicobacteraceae	Blank	4.91	4.53	0.021
p__Firmicutes.c__Clostridia.o__Oscillospirales.f__UCG-010	SSe	2.96	2.71	0.008
p__Actinobacteriota.c__Actinobacteria.o__Micrococcales.f__Micrococcaceae	Blank	2.24	2.95	0.010
p__Firmicutes.c__Bacilli.o__Erysipelotrichales.f__Erysipelotrichaceae.g__unclassified_f__Erysipelotrichaceae	SSe	2.56	2.57	0.037
p__Firmicutes.c__Clostridia.o__Monoglobales.f__Monoglobaceae.g__Monoglobus	Control	3.26	2.98	0.002
p__Proteobacteria.c__Alphaproteobacteria.o__Rhodospirillales.f__norank_o__Rhodospirillales.g__norank_f__norank_o__Rhodospirillales	SSe	3.44	3.15	0.029

p__Firmicutes.c__Clostridia.o__unclassified_c__Clostridia.f__unclassified_c__Clostridia.g__unclassified_c__Clostridia	SSe	1.82	3.22	0.015
p__Firmicutes.c__Clostridia.o__Peptostreptococcales-Tissierellales.f__Anaerovoracaceae.g__Eubacterium_nodatum_group	Control	3.17	2.94	0.003
p__Firmicutes.c__Clostridia.o__Christensenellales	SSe	1.93	2.96	0.016
p__Campilobacterota.c__Campylobacteria.o__Campylobacterales	Blank	4.91	4.53	0.021
p__Bacteroidota.c__Bacteroidia.o__Bacteroidales.f__Muribaculaceae.g__Muribaculum	SeMet	2.87	2.67	0.043
p__Firmicutes.c__Clostridia.o__Lachnospirales.f__Lachnospiraceae.g__ASF356	Control	3.79	3.48	0.012
p__Firmicutes.c__Clostridia.o__Peptostreptococcales-Tissierellales.f__Anaerovoracaceae.g__Family_XIII_AD3011_group	Control	2.94	3.02	0.000
p__Firmicutes.c__Clostridia.o__Lachnospirales.f__Lachnospiraceae.g__Lachnoclostridium	Control	3.96	3.60	0.010
p__Proteobacteria.c__Gammaproteobacteria.o__Burkholderiales.f__Sutterellaceae.g__Parasutterella	Control	3.20	2.97	0.002
p__Firmicutes.c__Clostridia.o__Lachnospirales.f__Lachnospiraceae.g__A2	Control	3.28	2.97	0.002
p__Firmicutes.c__Clostridia.o__Oscillospirales.f__Ruminococcaceae.g__Ruminococcus	SSe	3.44	3.18	0.013
p__Bacteroidota.c__Bacteroidia.o__Bacteroidales.f__Tannerellaceae.g__Parabacteroides	SeMet	3.84	3.46	0.024
p__Proteobacteria.c__Alphaproteobacteria.o__Rhodospirillales	SSe	3.44	3.15	0.029
p__Proteobacteria.c__Gammaproteobacteria.o__Burkholderiales.f__Sutterellaceae	Control	3.20	2.97	0.002
p__Firmicutes.c__Clostridia.o__Monoglobales.f__Monoglobaceae	Control	3.26	2.98	0.002
p__Proteobacteria.c__Gammaproteobacteria.o__Pseudomonadales	SeMet	2.65	2.70	0.007
p__Firmicutes.c__Clostridia.o__Lachnospirales.f__Lachnospiraceae.g__Eubacterium_xylanophilum_group	Blank	3.51	3.28	0.005
p__Firmicutes.c__Clostridia.o__Clostridiales.f__Clostridiaceae	SSe	2.65	2.56	0.008
p__Campilobacterota.c__Campylobacteria	Blank	4.91	4.53	0.021
p__Bacteroidota.c__Bacteroidia.o__Bacteroidales.f__Rikenellaceae.g__norank_f__Rikenellaceae	Blank	2.42	2.70	0.014
p__Firmicutes.c__Clostridia.o__Peptostreptococcales-Tissierellales.f__Anaerovoracaceae	Control	3.44	3.08	0.001

p__Actinobacteriota.c__Actinobacteria.o__Micrococcales.f__Micrococcaceae.g__Arthrobacter	Blank	2.23	2.99	0.038
p__Firmicutes.c__Clostridia.o__Oscillospirales.f__Butyricicoccaceae	Control	3.43	3.12	0.001
p__Actinobacteriota.c__Coriobacteriia.o__Coriobacteriales.f__Eggerthellaceae.g__unclassified_f__Eggerthellaceae	Blank	2.30	3.25	0.015
p__Firmicutes.c__Clostridia.o__Peptostreptococcales-Tissierellales.f__Anaerovoracaceae.g__Anaerovorax	SSe	2.01	3.22	0.002
p__Proteobacteria.c__Alphaproteobacteria.o__Rhodospirillales.f__norank_o__Rhodospirillales	SSe	3.44	3.15	0.029
p__Actinobacteriota.c__Actinobacteria.o__Micrococcales	Blank	2.36	2.92	0.002
p__Campilobacterota.c__Campylobacteria.o__Campylobacterales.f__Helicobacteraceae.g__Helicobacter	Blank	4.91	4.53	0.021
p__Proteobacteria.c__Gammaproteobacteria.o__Enterobacterales.f__Enterobacteriaceae.g__Klebsiella	Control	3.40	3.13	0.026
p__Firmicutes.c__Clostridia.o__Lachnospirales.f__Lachnospiraceae	Control	5.24	4.78	0.020
p__Firmicutes.c__Clostridia.o__Oscillospirales.f__Ruminococcaceae.g__Harryflintia	SSe	2.52	2.78	0.019
p__Firmicutes.c__Clostridia.o__unclassified_c__Clostridia	SSe	1.82	3.21	0.015
p__Proteobacteria.c__Gammaproteobacteria.o__Burkholderiales.f__Burkholderiaceae.g__Burkholderia-Caballeronia-Paraburkholderia	SSe	2.44	2.88	0.000
p__Firmicutes.c__Clostridia.o__Clostridiales	SSe	2.65	2.56	0.008
p__Proteobacteria.c__Gammaproteobacteria.o__Burkholderiales.f__Burkholderiaceae	SSe	2.44	2.92	0.003
p__Bacteroidota.c__Bacteroidia.o__Bacteroidales.f__Tannerellaceae	SeMet	3.84	3.46	0.024
p__Firmicutes.c__Clostridia.o__Lachnospirales.f__Lachnospiraceae.g__Lachnospiraceae_UCG-010	Control	1.96	3.27	0.013
p__Firmicutes.c__Clostridia.o__Monoglobales	Control	3.26	2.98	0.002
p__Firmicutes.c__Clostridia.o__Lachnospirales	Control	5.24	4.78	0.020
p__Proteobacteria.c__Gammaproteobacteria.o__Enterobacterales.f__Enterobacteriaceae.g__Escherichia-Shigella	Blank	4.54	4.22	0.041
p__Campilobacterota	Blank	4.91	4.53	0.021
p__Firmicutes.c__Bacilli.o__Lactobacillales.f__Streptococcaceae.g__Lactococcus	Blank	3.86	3.63	0.010
p__Firmicutes.c__Clostridia.o__Christensenellales.f__Christensenellaceae	SSe	1.93	2.95	0.016

p__Firmicutes.c__Clostridia.o__Oscillospirales.f__UCG-010.g__norank_f__UCG-010	SSe	2.96	2.71	0.008
